# Prediabetes Associates With Musculoskeletal Alterations Independent of Total Body Adiposity

**DOI:** 10.1002/jcsm.70198

**Published:** 2026-01-29

**Authors:** Alan Fappi, Clifton J. Holmes, Chao Cao, Vasavi Shabrish, Aman P. Aher, Karen Shen, Paul K. Commean, Dwight A. Towler, Dominic N. Reeds, Gretchen A. Meyer, Bettina Mittendorfer

**Affiliations:** ^1^ Department of Medicine Washington University School of Medicine Saint Louis Missouri USA; ^2^ Departments of Medicine and Nutrition & Exercise Physiology, School of Medicine University of Missouri Columbia Missouri USA; ^3^ Program in Physical Therapy Washington University School of Medicine Saint Louis Missouri USA; ^4^ Mallinkrodt Institute of Radiology Washington University School of Medicine Saint Louis Missouri USA

**Keywords:** fatigability, muscle, obesity, sarcopenia, weakness

## Abstract

**Background:**

Excess adiposity is a major risk factor for insulin resistance, prediabetes, and Type 2 diabetes and increases the risk for sarcopenia and osteosarcopenia later in life. It has been proposed that altered metabolic function and musculoskeletal status in people with obesity are directly linked, presumably because they share common pathophysiological mechanisms. However, the effect of metabolic dysfunction, independent of adiposity, on musculoskeletal status is unknown.

**Methods:**

We performed a comprehensive assessment of musculoskeletal status in people with overweight/obesity and prediabetes (*n* = 12; 72% women; age: 67 ± 6 years; weight: 81 ± 11 kg; mean ± SD) and a control group of sex‐, age‐ and adiposity‐matched participants with normoglycaemia (*n* = 18; 67% women; age: 65 ± 6 years; weight: 81 ± 12 kg).

**Results:**

Appendicular muscle mass expressed relative to the sarcopenia threshold (−5.6% ± 2.5% vs. 1.8% ± 2.0%; mean ± SEM) and the bone mineral density T‐score (−0.22 ± 0.41 vs. 0.82 ± 0.33) were lower (*p* < 0.05) in the prediabetic group than the control group. Additionally, the prediabetic group had ~25% smaller (by cross‐sectional area) myofibres and ~40% fewer muscle Type 2 macrophages (all *p* < 0.05), whereas intramyocellular lipid content was more than 50% higher (*p* < 0.05) in the prediabetic than the control group. Maximal muscle strength was not different between the two groups, but muscle strength during repeated maximum voluntary contractions declined more (*p* < 0.05) in the prediabetic group.

**Conclusion:**

In people with overweight/obesity, metabolic dysfunction associates with musculoskeletal dysfunction independent of adiposity.

## 1 Introduction

Excess body fat is associated with an increased risk of age‐associated sarcopenia (muscle depletion in conjunction with muscle weakness) and osteosarcopenia (sarcopenia in conjunction with osteopenia/osteoporosis) [1, 2] and a concomitant 2‐ to 3‐fold increased risk of falls and bone fractures, mobility disability, loss of independence and mortality [3, 4]. ‘Sarcopenic obesity’ and ‘osteosarcopenic obesity’ affect about 10%–30% of adults over the age of 50 years old with rising incidence [1, 2]. The mechanisms linking obesity with sarcopenia and osteosarcopenia are unclear. Our overarching hypothesis is that obesity‐associated metabolic alterations—in particular insulin resistance—rather than excess body fat per se adversely affect the musculoskeletal system. Insulin resistance is a key feature of prediabetes/Type 2 diabetes and closely associates with and possibly drives other abnormalities, including body fat redistribution to intra‐abdominal and ectopic fat depots, poor tissue perfusion, mitochondrial dysfunction and low‐grade, non‐infectious systemic inflammation [5], all of which are putative pathophysiological mechanisms involved in sarcopenia and osteosarcopenia [6, 7].

The primary goal of our study was to evaluate the effect of prediabetes, independent of adiposity, on skeletal muscle mass, bone mineral density, muscle strength and muscle fatigability. To this end, we measured appendicular lean/muscle mass and bone mineral density by using dual‐energy X‐ray absorptiometry, maximal handgrip strength and maximal upper and lower leg muscle strength by using a dynamometer, and the decline in maximal upper and lower leg muscle strength during repeat contractions (muscle fatigability) in people with overweight/obesity and prediabetes and a sex‐, age‐ and adiposity‐matched control group of participants with normoglycaemia. We hypothesized that appendicular lean/muscle mass and bone density scores relative to pre‐sarcopenia/sarcopenia and osteopenia/osteoporosis thresholds would be lower in the prediabetic group compared with the control group. Furthermore, we hypothesized that muscle would be weaker and fatigue more in the prediabetic group compared with the control group. Additionally, we assessed a series of secondary outcomes, including a panel of circulating markers of musculoskeletal status and putative muscle factors that can affect muscle mass and force generation, including muscle composition (by using magnetic resonance imaging and by histological analysis of muscle biopsy samples), muscle oxygen consumption (ex vivo in muscle biopsy samples) and the muscle expression of a priori selected representative key genes that are associated with insulin signalling and glycolysis, muscle growth, autophagy and inflammation. We also surveyed the muscle transcriptome (by using RNAseq) and the muscle proteome (by using LC–MS) to evaluate global alterations in muscle proteostasis.

## 2 Methods

### 2.1 Study Participants and Participant Flow

Thirty sedentary men and women with overweight/obesity (18 with normoglycaemia, 12 with prediabetes) participated in this study, which represents a secondary analysis of baseline data that was collected as part of an interventional clinical trial that was terminated early due to the COVID‐19 pandemic. The trial was approved by the Institutional Review Board at Washington University and was registered on Clinicaltrials.gov (NCT05145452). Participants provided written informed consent before initiating the study and completed a series of screening assessments, including a medical history and physical examination, standard blood tests and an oral glucose tolerance test to determine eligibility. Participants were included in the normoglycaemic control group if they had a plasma glucose concentration < 100 mg/dL after an overnight fast and < 140 mg/dL at 2 h after ingesting 75 g of glucose [8]. Participants were included in the prediabetic group if they had impaired glucose tolerance (plasma glucose ≥ 140 mg/dL but < 200 mg/dL at 2 h after ingesting 75 g of glucose) in conjunction with normal (< 100 mg/dL) or impaired (≥ 100 mg/dL but < 126 mg/dL) fasting plasma glucose [8]. We focused on participants with impaired glucose tolerance alone or impaired glucose tolerance in conjunction with impaired fasting plasma glucose because of the following: (i) isolated impaired fasting plasma glucose is uncommon in people with obesity [8] and (ii) impaired glucose tolerance is a robust marker of insulin resistance whereas impaired fasting glucose is primarily a measure of impaired beta‐cell function [8, 9].

Potential participants were excluded if they (i) were < 55 or > 80 years old; (ii) had a body mass index < 25.0 or ≥ 40.0 kg/m^2^; (iii) had signs or history of major organ system dysfunction or were taking medications or dietary supplements that could affect the study outcome measures or are incompatible with the testing procedures (e.g., certain metal implants); (iv) had a ≥ 3% body weight change within the prior 6 months; (v) participated in moderate or strenuous physical activities or structured exercise for more than 90 min per week; (vi) regularly consumed excessive amounts of alcohol (14 units per week for men, 7 units per week for women); or (vii) regularly smoked cigarettes. Nineteen participants of the total of 49 participants who were screened did not meet the inclusion criteria. After completing the screening visit, participants completed three testing visits to (i) evaluate body composition, (ii) conduct muscle function testing and (iii) obtain the muscle biopsy. Participants were instructed to consume their habitual diet and abstain from alcohol and strenuous physical activities for 3 days before each of the testing visits.

### 2.2 Body Composition

Dual‐energy X‐ray absorptiometry (Lunar iDXA with CoreScan, GE Healthcare) was used to evaluate total body and intra‐abdominal fat masses, total body fat‐free mass, appendicular lean mass (which provides an index of total appendicular skeletal muscle mass and is used to define presarcopenia/sarcopenia) and total body bone mass and bone mineral density. Magnetic resonance imaging (3T Magnetom Vida; Siemens) in conjunction with a custom‐made Matlab image analysis program was used to quantify thigh and calf muscle and inter‐ and intra‐muscular adipose tissue volumes. Imaging data represent the sum of nine sequential 5‐mm thick images each centred on the middle between the pubic symphysis and the medial knee joint space and the bottom of the gastrocnemius and the lateral knee joint space, respectively.

### 2.3 Muscle Strength and Fatigability

Handgrip strength of the dominant hand (average value of three repetitions) was evaluated by using a handheld dynamometer (Jamar, Duluth, MN). Upper and lower leg strength were evaluated by using a Biodex Medical Systems (Shirley, NY) 4 Pro dynamometer. We assessed unilateral (dominant leg) isometric (5 s‐long contraction) and isokinetic (60°/s) maximal voluntary contraction (MVC) torque and time to peak torque during knee extension, knee flexion and ankle dorsi‐ and plantarflexion exercises. Three repetitions each with a two‐minute rest period in between were performed and the average of the three values was used for analysis. The decline in torque (fatigability) during isokinetic (60°/s) knee extension and ankle plantar flexion was evaluated during 50 successive MVC with passive return. Torque variability during the repeated contraction protocol, which evaluates the ‘reliability’ of muscle power outputs during the repeated contraction test, was evaluated by using the squared residuals of the regression model that describes the peak torque time course during this test [10].

### 2.4 Muscle Biopsy Procedure, Histology, Respirometry, mRNA and Protein Expression

A muscle biopsy from the gastrocnemius medialis was obtained during local anaesthesia after participants fasted overnight and rested on a bed for at least 1 h. An aliquot of the fresh tissue sample was used to evaluate muscle oxygen consumption by using high‐resolution respirometry (O2k, Oroboros Instruments, Innsbruck, Austria) as described in [11, 12] with the following substrates: malate, glutamate, pyruvate (to measure complex I LEAK [CI_Leak_]), ADP (to measure complex I oxidative phosphorylation capacity [CI_oxphos_]), succinate (to measure complex I and II oxidative phosphorylation capacity [CI + II_oxphos_]), FCCP (to measure electron transport system capacity [ETS]) and rotenone (to inhibit oxidation [ETS_CI_]). Another portion of the fresh tissue sample was prepared for histology and immunostained to evaluate myofibre Type (1, 2a and 2x), fibre cross‐sectional area, myonuclei (DAPI+) content, capillary (lectin) density and macrophage (CD68/CD206) infiltration [13, 14]. Intramyocellular lipid content and collagen/fibrosis area were quantitated by Oil Red O and Picro Sirius Red staining, respectively [13]. A third portion of the tissue sample was snap frozen in liquid nitrogen and stored at −80°C for gene expression (RNAseq) and proteome analyses. For RNAseq analysis, total RNA was isolated from frozen tissue samples by using QIAzol lysis reagent and a RNeasy Fibrous Tissue mini kit (#74704Qiagen, Valencia, CA) in combination with TRIzol/Choloroform (#15‐596‐018, Invitrogen, Waltham, MA). RNA integrity was confirmed with an RNA integrity number (RIN value) > 7 and libraries were prepared by using a TruSeq Stranded mRNA kit (#20020594, Illumina, San Diego, CA) with total RNA and cDNA fragments generated on an Illumina NovaSeq‐PE‐100 6000. Raw sequencing data were analysed by using Fastp and STAR to quantify mapped reads and prepare a gene count matrix. The DESeq2 software in R was used to normalize the counts and the counts per million (CPM) reads were used for further analysis. For proteomic analysis, muscle proteins were precipitated with acetone, digested with LysC and trypsin, and the peptides were analysed by LC–MS (Bruker nanoElute system attached to a Bruker timsTOF‐PRO mass spectrometer via a Bruker CaptiveSpray source). D‐PASEF data were collected and searched using Bruker PaSER, employing the inbuilt Human spectral library and default parameters [15].

### 2.5 Circulating Biomarkers of Musculoskeletal Status

The concentrations of a panel of biomarkers (myostatin, follistatin, brain‐derived neurotrophic factor, N‐terminal Type III procollagen, insulin‐like growth factor 1, dehydroepiandrosterone sulphate, cortisol, C‐reactive protein, interleukin‐6, tumour necrosis factor‐α, creatinine, cystatin C, osteocalcin, C‐terminal telopeptide of Type I collagen, alkaline phosphatase, bone‐specific alkaline phosphatase) that has been endorsed by the World Health Organization for musculoskeletal conditions [16] was evaluated by using commercially available ELISA kits (Thermo Scientific KAQ1381, EIADHEA; ABCAM ab285249; MyBiosource MBS285496; Biomatik 50‐150‐0042; and Novus Biologicals NBP2‐76434) and immunoplex assay (Thermo Scientific PPX‐04, EPX010‐12279‐901, EPX01A‐10288‐901, HMYOMAG‐56K, EPX010‐12244‐901, EPX010‐10420‐901).

### 2.6 Definitions of Sarcopenia, Presarcopenia, Osteoporosis and Osteopenia

Sarcopenia was defined as an appendicular muscle mass value (kg) below a sex‐specific threshold [i.e., less than −13.19 + 14.75 × height (in meters) + 0.23 × total fat mass (in kg) for women and less than −22.48 + 24.14 × height (in meters) + 0.21 × total fat mass (in kg) for men [17]), in conjunction with low grip strength in the dominant hand (< 20 kg for women and < 30 kg for men) [3, 17, 18]. Pre‐sarcopenia was defined as low appendicular muscle mass alone [3, 18]. Osteopenia was defined as a T‐score less than −1.0 but greater than −2.5; osteoporosis was defined as a T‐score equal to or less than −2.5 [4, 7].

### 2.7 Statistical Analysis

One‐ and two‐way analysis of variance (Prism 10.2.0, GraphPad) was used as appropriate to evaluate differences in participant characteristics and outcome variables between the prediabetic and control groups. Skewed data sets were log‐transformed to achieve normality before analysis. Differences in the prevalence of presarcopenia/sarcopenia and osteopenia/osteoporosis between groups were evaluated by using the Χ^2^ test. Multiple linear regression analysis was used to evaluate the relationship between insulin sensitivity, skeletal muscle morphological characteristics and muscle fatigue. A *p* value ≤ 0.05 was considered statistically significant. Functional enrichment analysis of the RNAseq and proteome data was performed by using g:Profiler, a public web service (https://biit.cs.ut.ee/gprofiler; 2023 update) that is an ELIXIR recommended interoperability resource with an R‐package interface [19]. Canonical pathways with an experiment‐wide threshold of *p* = 0.05 (i.e., at least 95% of matches above threshold)—evaluated by using the g:SCS algorithm [19]—were considered statistically significant. Data are expressed as mean ± SEM unless otherwise noted.

## 3 Results

### 3.1 Participant's Sex, Age, Glycaemic Status, Body Composition and Global Musculoskeletal Status

There were no differences in age, total body mass, body mass index and the contribution of total body fat mass to total body mass between the prediabetic and the control groups (Table 1A). About two‐thirds of the participants in both groups were women (Table 1A). By design, oral glucose tolerance was markedly impaired in the prediabetic group compared with the control group, whereas fasting plasma glucose concentration was not different between the groups (Table 1A, Figure S1). Fasting plasma insulin and the HOMA insulin resistance index were higher, and the Matsuda insulin sensitivity index was lower in the prediabetic group compared with the control group (Table 1A). Appendicular lean mass, expressed in kg, and handgrip strength were not different between the prediabetic and the control groups (Table 1A). However, appendicular lean mass in relationship to the sex‐ and adiposity‐adjusted lean mass value that is used to assess pre‐sarcopenia and sarcopenia was lower in the prediabetic group, and more participants in the prediabetic group had an appendicular lean mass value below the sarcopenia threshold (Table 1A). About 75% of participants in the prediabetic group and 33% of participants in the control group had presarcopenia (low appendicular muscle mass value), and about 10% of participants in both groups had sarcopenia (low appendicular muscle mass value in conjunction with low grip strength) (Table 1A). Bone mineral density, expressed as g/cm^2^, was not different between the prediabetic and the control groups, but the bone mineral density T‐score was lower in the prediabetic group, and more participants in the prediabetic group had a T‐score in the osteopenia range (Table 1A). None of the participants had osteoporosis.

**TABLE 1:** Participants' age, body composition and metabolic and global musculoskeletal status.

	Control	Prediabetes
A: Participants' age, body composition and metabolic and global musculoskeletal status		
*N* (women, men)	18 (13, 5)	12 (8, 4)
Age (years)	65 ± 6	67 ± 6
Fasting plasma insulin (mU/L)	7.9 ± 2.9	12.0 ± 7.1*
Fasting plasma glucose (mg/dL)	92 ± 5	95 ± 9
2‐h OGTT plasma glucose (mg/dL)	110 ± 18	157 ± 21*
HOMA insulin resistance index	1.8 ± 0.7	2.9 ± 1.9*
Matsuda insulin sensitivity index	4.4 ± 1.9	2.9 ± 1.4*
Height (cm)	165 ± 9	166 ± 8
Body mass index (kg/m^2^)	30 ± 4	30 ± 3
Total body mass (kg)	81 ± 12	81 ± 11
Body fat (% total body mass)	40 ± 7	42 ± 7
Fat mass, total body (kg)	33 ± 7	34 ± 8
Intra‐abdominal fat mass (kg)	1.3 ± 0.8	1.7 ± 0.8*
Fat‐free mass, total body (kg)	49 ± 10	47 ± 8
Appendicular lean mass (kg)	21 ± 5	20 ± 4
Appendicular lean mass relative to sarcopenia threshold (%)^a^	1.8 ± 8.5	−5.6 ± 8.7*
Appendicular lean mass below sarcopenia threshold (n, %)^a^	8 (44%)	10 (83%)*
Presarcopenia (*n*, %)^b^	6 (33%)	9 (75%)
Sarcopenia (*n*, %)^c^	2 (11%)	1 (8%)
Bone mineral mass (kg)	2.5 ± 0.5	2.4 ± 0.5
Bone mineral density (g/cm^2^)	1.20 ± 0.16	1.10 ± 0.16
Bone mineral density (T‐score)	0.82 ± 1.42	−0.22 ± 1.42*
Osteopenia (*n*, %)^d^	1 (6%)	4 (33%)*
Maximum handgrip strength (kg)	30 ± 9	29 ± 9
B: Circulating biomarkers of musculoskeletal status		
Insulin‐like growth factor 1 (μg/L)	47 ± 12	38 ± 12*
Myostatin (ng/L)	360 [164, 688]	452 [229, 946]
Follistatin (ng/L)	211 [162, 264]	207 [150, 441]
Brain‐derived neurotrophic factor (ng/L)	1.68 [0.50, 5.16]	1.84 [0.41, 5.40]
N‐terminal type III procollagen (ng/L)	718 ± 420	593 ± 360
Dehydroepiandrosterone sulphate (μg/L)	956 ± 803	974 + 581
Cortisol (μg/L)	606 ± 669	764 ± 909
C‐reactive protein (μg/)	2392 ± 1925	3231 ± 1380
Interleukin 6 (ng/L)	1.31 ± 0.94	1.57 ± 1.21
Tumour necrosis factor alpha (ng/L)	0.14 [0.04, 0.28]	0.13 [0.06, 0.25]
Cystatin C (mg/L)	0.16 ± 0.03	0.15 ± 0.02
Creatinine (mg/L)	8.9 ± 1.9	8.1 ± 1.8
Osteocalcin (μg/L)	15.4 ± 7.7	11.1 ± 3.7*
Cross‐linked C‐telopeptide of Type I collagen (μg/L)	58 [40, 80]	60 [42, 68]
Alkaline phosphatase (μg/L)	6.53 [0.49, 20.81]	8.77 [1.57, 20.46]
Bone‐specific alkaline phosphatase (μg/L)	2.59 ± 1.32	2.54 ± 1.37
C: Maximal leg muscle strength and leg muscle volume and composition		
Isometric knee extension		
Peak torque (Nm)	108 ± 38	110 ± 33
Time to peak (s)	3.1 ± 1.1	3.1 ± 0.8
Isokinetic (60°/s) knee extension		
Peak torque (Nm)	70 ± 23	72 ± 34
Time to peak (s)	0.7 ± 0.3	1.0 ± 0.8
Isometric dorsiflexion		
Peak torque (Nm)	21 ± 13	19 ± 13
Time to peak (s)	1.8 ± 1.0	1.4 ± 0.7
Isokinetic (60°/s) dorsiflexion		
Peak torque (Nm)	16 ± 6	16 ± 6
Time to peak (s)	0.8 ± 0.6	0.9 ± 0.4
Isometric plantarflexion		
Peak torque (Nm)	66 ± 28	69 ± 23
Time to peak (s)	4.0 ± 0.9	3.2 ± 1.2
Isokinetic (60°/s) plantarflexion		
Peak torque (Nm)	39 ± 20	43 ± 12
Time to peak (s)	0.7 ± 0.1	0.7 ± 0.1
Thigh muscle volume (cm^3^)	480 ± 135	413 ± 121
Thigh intermuscular adipose tissue volume (cm^3^)	72 ± 9	60 ± 8
Thigh intramuscular adipose tissue volume (cm^3^)	2.3 ± 0.9	1.5 ± 0.5
Calf muscle volume (cm^3^)	213 ± 81	226 ± 93
Calf intermuscular adipose tissue volume (cm^3^)	12.8 ± 8.8	12.0 ± 5.9
Calf intramuscular adipose tissue volume (cm^3^)	2.6 ± 2.1	1.9 ± 1.3

*Note:* Data are mean ± SD or median [interquartile range].

^a^ The appendicular lean mass threshold used to define sarcopenia was calculated as −13.19 + 14.75 × height (in meters) + 0.23 × total fat mass (in kg) for women and less than −22.48 + 24.14 × height (in meters) + 0.21 × total fat mass (in kg) for men [3, 17, 18].

^b^ Presarcopenia was defined as low appendicular muscle mass alone [3, 18].

^c^ Sarcopenia was defined as an appendicular muscle mass value below the threshold in conjunction with low grip strength in the dominant hand (< 20 kg for women and < 30 kg for men) [3, 17, 18].

^d^ Osteopenia was defined as a T‐score less than −1.0 but greater than −2.5; osteoporosis was defined as a T‐score equal to or less than −2.5 [4, 7]. None of the participants in this study had osteoporosis.

* Value significantly different from the value in the control group, *p* ≤ 0.05.

### 3.2 Circulating Biomarkers of Musculoskeletal Health

Among the 16 plasma biomarkers that have been endorsed by the World Health Organization for musculoskeletal conditions [16], the concentrations of two (insulin‐like growth factor 1, osteocalcin) were lower in the prediabetic compared with the control group (Table 1B); the concentrations of the other proteins were not different between the groups (Table 1B).

### 3.3 Leg Muscle Strength

**FIGURE 1:**
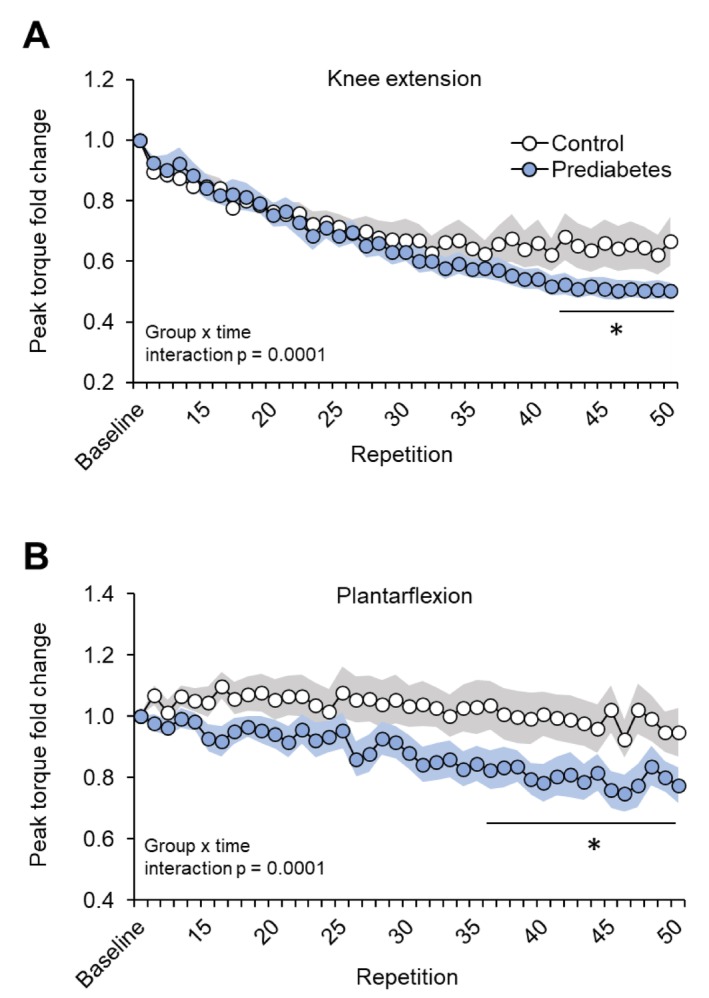
Change in maximal voluntary contraction torque during 50 sequential maximum voluntary contractions in the prediabetic group and the normoglycaemic control group. Maximum voluntary contraction torque decline during repeated isokinetic (60°/s) knee extensions (A) and plantar flexions (B) in the prediabetic group (*n* = 12) and the control group (*n* = 18). Values are mean values (circles) ± SEM (shaded areas). Baseline values represent the average value of five contractions. ANOVA revealed a significant group × time interaction. Post hoc analysis revealed significant differences between groups starting at repetition 42 for knee extension and repetition 36 for plantarflexion.

Maximum voluntary isometric and isokinetic (60°/s) knee extensor and plantar and dorsiflexion strength (MVC torque) were not different between the prediabetic and control groups (Table 1C), but the decline in strength during repeated isokinetic (60°/s) knee extension and plantar flexion was greater in the prediabetic group than the control group (Figure 1). Torque variability was not different between groups during knee extension [28.8 (22.1, 47.1) and 28.2 (19.9, 36.7); median (quartiles)] or plantar flexion [5.1 (3.2, 8.2) and 6.6 (3.2, 7.4); median (quartiles)].

### 3.4 Leg Muscle Volume and Composition and Muscle Histology

**FIGURE 2:**
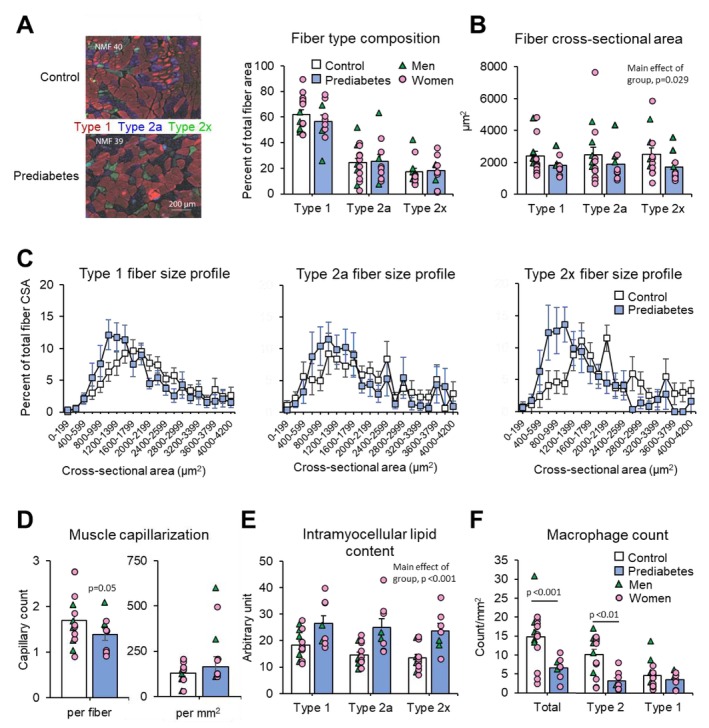
Gastrocnemius histology in the prediabetic and control groups. (A) Representative fibre‐type stains from one participant in the control group and one participant in the prediabetic group (left) and fibre type composition, expressed as Types 1, 2a and 2x fibre cross‐sectional areas as proportion of total fibre cross‐sectional area (right). B: Average Type 1, Type 2a and Type 2x fibre cross‐sectional area. (C) Type 1, Type 2a and Type 2x fibre size profile. (D) Myofibre and muscle capillarization assessed by lectin staining. Data are expressed as capillary count per fibre and capillary count per mm^2^ cross‐sectional area. (E) Intramyocellular lipid content, assessed by Oil Red O staining. (F) Type 1 and Type 2 macrophage count, assessed by CD68 and CD206 staining. Values are mean ± SEM with individual data shown in circles (women) and triangles (men) for the prediabetic group and the control group.

There were no differences between the prediabetic and control groups in leg muscle volume and inter‐ and intra‐muscular adipose tissue contents evaluated by using magnetic resonance imaging (Table 1C), the relative contributions of Type 1, Type 2a and Type 2x fibres to total fibre cross‐sectional area evaluated by histology (Figure 2A) and collagen content evaluated by histology (0.12 ± 0.01 vs. 0.13 ± 0.01 area fraction). However, the prediabetic group had smaller myofibres (by cross‐sectional area) (Figures 2B,C). Myonuclei content per fibre was also lower in the prediabetic compared with the control group in both Type 1 (1.2 ± 0.2 vs. 2.2 ± 0.3, *p* < 0.01) and Type 2 (1.3 ± 0.3 vs. 2.1 ± 0.3, *p* < 0.05) fibres, but myonuclear domain size was not different between the two groups in either Type 1 (4629 ± 394 and 4092 ± 330 μm^2^) or Type 2 (4713 ± 552 and 4293 ± 393 μm^2^) fibres, because myofibres were smaller in the prediabetic group. Similarly, capillary count per fibre was about 25% less in the prediabetic group compared with the control group (Figure 2D), but capillary density (capillary count per cross‐sectional area) was not different between the prediabetic group and the control group (Figure 2D), because myofibre count per cross‐sectional area was greater in the prediabetic group due to the smaller myofibre size. Intramyocellular lipid content was about 50% higher in the prediabetic group compared with the control group (Figure 2E), and total macrophage content (assessed as CD68‐positive cells) was about 40% less in the prediabetic group due to a reduced number of Type 2 macrophages (CD206‐positive cells), whereas Type 1 macrophage (CD68‐positive/CD206‐negative cells) content was not different between the two groups (Figure 2F). In a multiple linear regression model that included the decline (slope) in plantarflexion torque as the dependent variable and intramyocellular lipid content, capillary count per fibre, macrophage content and the Matsuda insulin sensitivity index as independent variables, total and Type 2 macrophage contents emerged as significant (*p* < 0.05) predictors of muscle fatigue.

### 3.5 Muscle mRNA and Protein Expression

**FIGURE 3:**
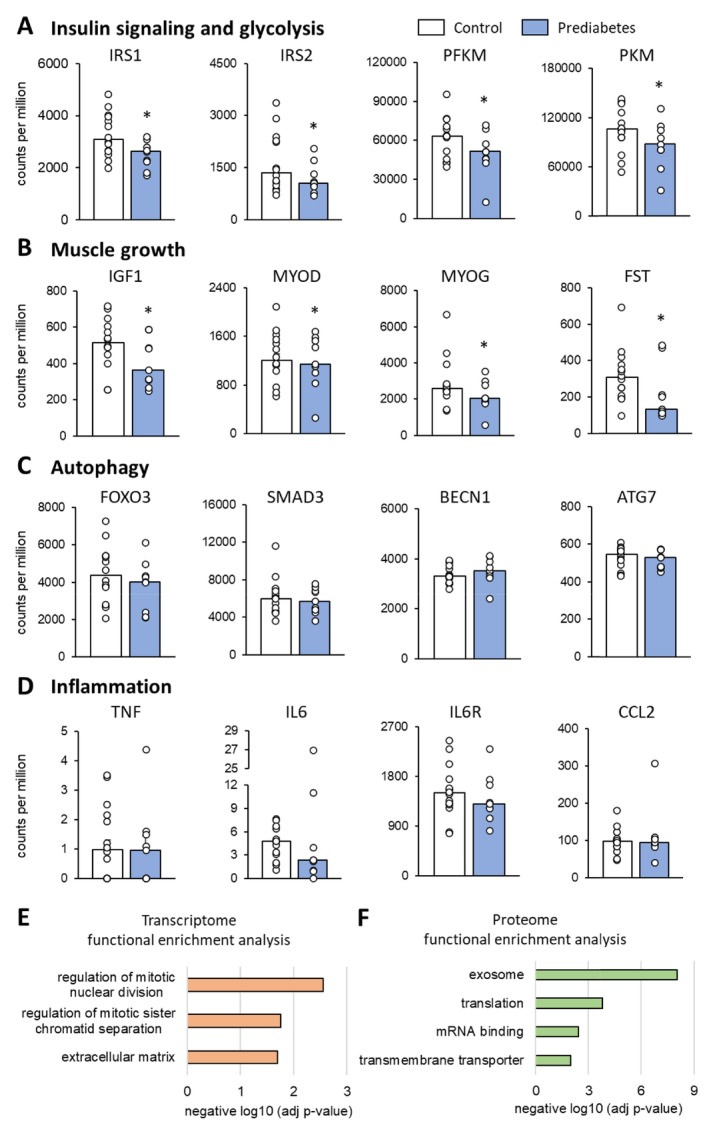
Muscle mRNA and protein expression. Panels (A–D): mRNA expression of key representative genes associated with insulin signalling and glycolysis (A), muscle growth (B), autophagy (C) and inflammation (D) in the prediabetic group and the control group. Values are mean ± SEM with individual data shown in circles. Panels (E–F): Functional enrichment analysis of the muscle transcriptome (E) and proteome (F). A list of key genes/proteins in the pathways shown here is provided in Table S1. Abbreviations: ATG7, autophagy related 7; BECN1, beclin 1; CCL2, c‐c motif chemokine ligand 2; FOXO3, forkhead box O3; FSTN, follistatin; IGF1, insulin‐like growth factor 1; IL6, interleukin 6; IL6R, interleukin 6 receptor; IRS1, insulin receptor substrate 1; insulin receptor substrate 2; MYOD, myogenic differentiation; MYOG, myogenin; PFKM, phosphofructokinase; PKM, pyruvate kinase muscle isozyme; SMAD3, mothers against decapentaplegic homologue 3; tumour necrosis factor.

Compared with the control group, the a priori selected genes involved in insulin signalling and glycolysis (IRS1, IRS2, PFKM, PKM) and muscle growth (IGF1, MYOD, MYOG, FSTN) were downregulated in the prediabetic group (*p* < 0.05) whereas the expression of the a priori selected genes involved in autophagy (FOXO3, SMAD3, BECN1, ATG7) and inflammation (TNF, IL6, IL6R, CCL2) was not different between the prediabetic and the control groups (Figure 3). The global muscle gene and protein expression survey found 328 of the more than 22 000 genes were ≥ 50% (log2 value = 0.58) upregulated (*n* = 196) or downregulated (*n* = 132) in the prediabetic compared with the control group (Figure S2) and 21 of the 2900 proteins monitored were ≥ 50% (log2 value = 0.58) upregulated (*n* = 9) or downregulated (*n* = 12) in the prediabetic compared with the control group (Figure S3). Functional enrichment analysis found group differences in the expression of mRNA sets related to cell division and extracellular matrix (Figure 3E and Table S1) and in the expression of protein sets related to extracellular vesicles/exosomes, translation, mRNA binding and transmembrane transporter activity (Figure 3F and Table S1).

### 3.6 Muscle Oxygen Consumption

Mitochondrial oxygen flux assessed ex vivo in muscle biopsy samples was higher in the prediabetic group than the control group (CI_Leak_: 11.1 ± 1.6 vs. 8.5 ± 0.6; CI_oxphos_: 51.8 ± 9.3 vs. 35.5 ± 5.1; CI + II_oxphos_: 97.2 ± 10.1 vs. 69.2 ± 5.9; ETS: 94.7 ± 12.9 vs. 71.9 ± 5.4; ETS_CI_: 59.9 ± 9.6 vs. 47.4 ± 3.7; all expressed as pmol/s per mg tissue; main effect of group, *p* < 0.05), but mitochondrial coupling efficiency, expressed as the respiratory control ratio (defined as CI + II_oxphos/_CI_Leak_ [11, 12]), was not different between the prediabetic group and the control group (9.9 ± 1.6 vs. 8.6 ± 0.5, respectively).

## 4 Discussion

We evaluated the association between metabolic dysfunction (defined as pre‐diabetes) and musculoskeletal status independent of total body adiposity in late middle‐aged and older adults with overweight/obesity. Although maximal muscle strength (assessed as maximal handgrip strength and voluntary isometric and isokinetic contraction torque in leg muscles) was not different between the prediabetic group and the control group, the decline in leg muscle strength during repeated maximal voluntary contractions was greater in the prediabetic group. In addition, appendicular muscle mass and bone mineral density scores, myofibre size and muscle Type 2 macrophage content were lower and intramyocellular lipid (but not inter‐ or intra‐muscular adipose tissue) content was higher in the prediabetic group compared with the control group. These data demonstrate an association between metabolic dysfunction and altered musculoskeletal status independent of adiposity in people with overweight/obesity.

Leg muscle volume assessed by using magnetic resonance imaging and appendicular lean/muscle mass assessed by using dual‐energy X‐ray absorptiometry were not different between the prediabetic and control groups. However, the appendicular lean/muscle mass score, which evaluates appendicular lean mass relative to height‐, fat mass‐ and sex‐adjusted standards [17], myofibre size (cross‐sectional area) and the expression of muscle growth genes were lower in the prediabetic group compared with the control group. Standardized data for magnetic resonance imaging‐based measures of muscle mass do not exist. Similarly, absolute bone mass and bone mineral density values were not different between the prediabetic and control groups but the bone mineral density values relative to normative osteopenia/osteoporosis values (T‐score) were lower in the prediabetic group. Nearly twice as many participants in the prediabetic group (83%), compared with the control group (44%) had suboptimal skeletal muscle status (i.e., a muscle mass score in the pre‐sarcopenic or sarcopenic range) and 33% of participants in the prediabetic group, compared with 6% of participants in the control group had osteopenia. These findings underscore the utility of normative data when evaluating musculoskeletal status. They also suggest that the unfavourable musculoskeletal status that is often observed in people with obesity compared with lean people [4] is likely due to, or possibly accelerated by insulin resistance and associated metabolic alterations that occur in many people with obesity, rather than or in addition to excess body fat per se.

We used a repeated MVC protocol to evaluate leg muscle contractile function, which allowed us to assess the maximal capacity for force production as well as the ability to sustain force production (fatigability). Although maximal handgrip strength and peak leg muscle MVC torque were not different between the prediabetic and control groups, suggesting no impairment in maximal force generation capacity in the prediabetic compared with the control group, the decline in MVC torque during repeated contractions of leg muscles was greater in the prediabetic group, suggesting muscle in people with prediabetes is more susceptible to fatigue. This finding is likely clinically relevant, because the ankle and knee joint motion torques involved during common physical activities (such as stair climbing, walking or running) require the maximal or near maximal dynamic voluntary contraction torque values we observed in our study participants [20, 21, 22]. Furthermore, the knee and ankle angular velocities we chose (60°/s) have ‘real life’ relevance. For example, mean knee angular velocity during chair rise (sit‐to‐stand transition) is ~60°/s [23]; and knee and ankle angular velocities involved in walking range from about 30°/s to peak values > 100°/s during slow and progressively faster walking [24, 25].

Muscle fatigue is caused by an intricate network of muscle intrinsic and extrinsic factors that include muscle energetics (phosphocreatine hydrolysis, glycolysis, mitochondrial oxidation), cross‐bridge cycling and the vascular and nervous systems. During short‐duration (< 2 min) repeated MVC, like in our study, muscle relies predominantly on hydrolysis of phosphocreatine and glycolysis for energy [26, 27] and about 75% of fatigue is due to muscle intrinsic factors, with the remaining 25% being due to waning neuronal drive [28, 29]. The expression of genes involved in glycolysis was downregulated in our prediabetic group compared with the control group, suggesting lower glycolytic capacity in the prediabetic group. Oxidative respiration, on the other hand, was most likely not impaired in the prediabetic group because muscle capillary density (capillary count per mm^2^ cross‐sectional area) and muscle oxygen flux were not reduced in the prediabetic group. This observation is in line with the results from other studies that found no relationship between dysregulated glucose metabolism/insulin resistance and in vivo or ex vivo assessments of mitochondrial function [30, 31, 32]. To better understand the mechanistic underpinnings of greater muscle fatigability in people with prediabetes, future studies can be designed to evaluate the effect of prediabetes/insulin resistance on muscle energetics in vivo, cross‐bridge cycling and neuronal drive.

We did not observe gross anatomical differences in skeletal muscle composition (inter‐ and intra‐muscular adipose tissue contents) between the prediabetic and control groups, but intramyocellular lipid content was about 1.5‐fold higher in the prediabetic group. Excess accumulation of intramyocellular lipids, a sign of an imbalance between myocellular lipid utilization and lipid synthesis and/or delivery, occurred in all three fibre types without a difference among fibre types. Additionally, we found myofibres were smaller (by cross‐sectional area) in the prediabetic group compared with the control group. We also found the expression of a set of a priori selected key genes related to muscle growth (including *IGF1*, *MYOD*, *MYOG*, *FSTN*) was lower in the prediabetic group than the control group and our transcriptome and proteome analyses identified differences in the translational program, which could be responsible for limiting myofibre size in people with prediabetes whereas genes/proteins related to muscle degradation appeared unaffected. On the other hand, there was no difference in myonuclear domain size between the prediabetic and control groups, suggesting the ‘nuclear capacity’ for muscle maintenance is not impaired in people with prediabetes. Myofibre type composition (i.e., the proportional contribution of different fibre types to total fibre cross‐sectional area) was not different between the prediabetic and control groups, ruling out fibre type composition as a factor involved in the between group difference in muscle fatigability. Furthermore, these data suggest that the difference in fibre type composition that has been observed between lean people and people with obesity [33, 34] is due to excess adiposity rather than metabolic dysfunction. The notion that obesity, not metabolic dysfunction, causes a shift in fibre type composition is supported by studies conducted in rats [35, 36].

Skeletal muscle macrophages represent a heterogeneous group of immune cells that can cause tissue inflammation but are also an integral component of the complex network of processes that regulate skeletal muscle mass and metabolic and contractile function [37]. Total muscle macrophage content was lower in the prediabetic group compared with the control group because of a marked reduction in Type 2 macrophages. Type 2 macrophages are anti‐inflammatory, mediate muscle growth and repair processes and increase in response to endurance exercise training [38]. The depletion of Type 2 macrophages in the prediabetic group might therefore be causally involved in the smaller myofibre size and possibly also greater fatigability in the prediabetic group. Future studies can interrogate this possibility. The mechanisms responsible for the lower Type 2 macrophage content in the prediabetic group are unclear and could involve reduced recruitment of circulating monocytes, increased senescence of resident macrophages, or both.

Our study has some limitations. First, the study design does not allow us to establish cause and effect or the direct mechanistic underpinnings for the relationship between metabolic dysfunction and alterations in musculoskeletal status. Most likely the relationship is bidirectional and may involve a vicious cycle whereby metabolic dysfunction fuels altered muscle function, which feeds metabolic dysfunction or vice versa. Secondly, the number of participants in our study is small and may not be sufficiently representative to draw general conclusions. Third, the results from our study demonstrate that body fat mass per se is not a driver of the musculoskeletal alterations we observed in people with prediabetes, but we cannot rule out potential crosstalk between dysfunctional adipose tissue in people with prediabetes and the musculoskeletal system. Future studies can be designed to interrogate the specific mediators of musculoskeletal alterations in people with prediabetes. Fourth, we did not evaluate mTOR signalling, which is a key growth regulator [39, 40]. Fifth, we collected a muscle biopsy from the gastrocnemius, which is relevant for the plantarflexion assessments in our study, but not the vastus lateralis, which would be relevant for the knee extension assessments. We limited the number of biopsies in our study in consideration of participant burden. Further, muscle biopsy analyses focus on a relatively small number of outcomes because of the small sample yield. Lastly, the gene and proteome expression analyses provide insight into the ‘whole muscle’ genome and proteome and cannot distinguish among different cell types within muscle.

In summary, the data from our study demonstrate that prediabetes is associated with alterations in musculoskeletal biology independent of total body adiposity. Although prediabetes does not affect maximum muscle strength, it is associated with greater fatigability during repeated contractions. This finding likely has important clinical implications, because it may make exercising—the frontline intervention to improve both metabolic and musculoskeletal function—more difficult. Although the precise causative mechanisms for the alterations in musculoskeletal status associated with prediabetes remain undefined, the data from our study point towards multiple putative targets that can be explored in future mechanistic studies.

## Author Contributions

B.M designed the study and supervised the performance of the study, sample and data processing, and interpretation of the results. A.F, C.J.H, C.C, V.S, A.P.A, K.S, P.K.C, D.A.T, D.N.R, and G.A.M assisted in conducting the clinical studies, sample and data processing, and interpretation of the results. A.F and B.M prepared the first draft of the manuscript. All authors critically reviewed and edited the manuscript. B.M is the guarantor of this work, had full access to all the data in the study, and assumes full responsibility for the integrity of the data and the accuracy of the data analysis.

## Funding

This study was supported by National Institutes of Health grants P30 DK056341 (Washington University Nutrition and Obesity Research Center), P30 DK020579 (Washington University Diabetes Research Center), P30 AR074992 (Washington University Musculoskeletal Research Center), UL1 TR002345 (Washington University Institute of Clinical and Translational Sciences), and R01 AR075773, and a grant from the Longer Life Foundation (2019‐011). The funding sources had no role in the design and conduct of the study; collection, management, analysis and interpretation of the data; preparation, review or approval of the manuscript; and decision to submit the manuscript for publication.

## Disclosure

This paper has not been presented in abstract form at a scientific conference or been submitted to a preprint server.

## Conflicts of Interest

The authors declare no conflicts of interest.

## Supporting information


**Table S1 related to Figure 3.** Functional enrichment analysis of the transcriptome and the proteome.
**Figure S1: related to Table 1.** Plasma glucose (left) and insulin (right) concentrations during the oral glucose tolerance test in the prediabetic group (*n* = 12) and the control group (*n* = 18). Values are mean ± SEM * Value significantly different from Control value, *p* < 0.01.
**Figure S2: related to Figure 3.** Volcano plot of differences in muscle mRNA expression between the prediabetic group and the control group. The top 30 (among a total of 132) downregulated genes and the top 30 (among a total of 196) upregulated genes are shown with their abbreviated names. A total of 22 419 genes were monitored and differential expression was defined as a log2 fold‐difference value ≥ 0.58 in conjunction with a ‐log10 *p* value ≥ 1.3.
**Figure S3: related to Figure 3.** Volcano plot of differences in muscle protein expression between the prediabetic group and the control group. A total of 2900 proteins were monitored and the significantly down‐ and upregulated proteins are shown with their abbreviated names. Differential expression was defined as a log2 fold‐difference value ≥ 0.58 in conjunction with a ‐log10 *p* value ≥ 1.3.
