# Supplementary material for: Prediabetes Associates With Musculoskeletal Alterations Independent of Total Body Adiposity
Source: J Cachexia Sarcopenia Muscle. 2026 Jan 29;17(1):e70198. doi: 10.1002/jcsm.70198 (PMC12856049; doi:10.1002/jcsm.70198)
Supplement: Supplementary file 1 — Table S1 related to Figure 3. Functional enrichment analysis of the transcriptome and the proteome. Figure S1: related to Table 1. Plasma glucose (left) and insulin (right) concentrations during the oral glucose tolerance test in the prediabetic group (n = 12) and the control group (n = 18). Values are mean ± SEM * Value significantly different from Control value, p < 0.01. Figure S2: related to Figure 3. Volcano plot of differences in muscle mRNA expression between the prediabetic group and the control group. The top 30 (among a total of 132) downregulated genes and the top 30 (among a total of 196) upregulated genes are shown with their abbreviated names. A total of 22 419 genes were monitored and differential expression was defined as a log2 fold‐difference value ≥ 0.58 in conjunction with a ‐log10 p value ≥ 1.3. Figure S3: related to Figure 3. Volcano plot of differences in muscle protein expression between the prediabetic group and the control group. A total of 2900 proteins were monitored and the significantly down‐ and upregulated proteins are shown with their abbreviated names. Differential expression was defined as a log2 fold‐difference value ≥ 0.58 in conjunction with a ‐log10 p value ≥ 1.3. [file JCSM-17-e70198-s001.docx]

**SUPPLEMENTAL MATERIAL**

**Prediabetes associates with musculoskeletal alterations independent of total body adiposity**

Alan Fappi, Clifton J. Holmes, Chao Cao, Vasavi Shabrish, Aman P. Aher, Karen Shen, Paul K. Commean, Dwight A. Towler, Dominic N. Reeds, Gretchen A. Meyer, Bettina Mittendorfer

| **Supplemental Table 1 related to Figure 3.** Functional enrichment analysis of the transcriptome and the proteome | | |
| --- | --- | --- |
| GO term and name | adj. p-value | Key genes |
| Transcriptome |  |  |
| GO:0007088 Regulation of mitotic nuclear division | 0.0028 | KNL1, CDC25C, DLGAP5, MKI67, TTK, BIRC5, AURKB |
| GO:0010965 Regulation of mitotic sister chromatid separation | 0.0176 | KNL1, DLGAP5, TTK, BIRC5, AURKB |
| GO:0031012 Extracellular matrix | 0.0201 | MARCO, PRG2, POSTN, C1QC, F13A1, MXRA5, COL8A2, PRG4, ANGPTL7, EMILIN2, VCAN |
| Proteome |  |  |
| GO:0070062 extracellular exosome | <0.00001 | NAPG, LDHB, SMS, CRYZ, PCBP2, GNPDA1, SYPL1, TOMM70, PCBP1, AGRN, EIF6, ANP32B, MSRA, CBR1, SERPINA7, CFD, ANO6, CFL2, MYH11, PLCB1, BCAM, EIF3E, PLD3, NCKAP1, SERPINB6, CANX, GHITM, HLA-DRB1, LAMA5, RPS13, H2AZ1, H2AZ2 |
| GO:0006412 translation | <0.001 | RPLP1, MRPL30, EIF4E3, TARS3, EIF6, EIF3J, RPS21, FARSA, CARS1, RCC1L, HNRNPD, EIF3E, CNBP, TBCE, EIF3F, RPS13 |
| GO:0003729 mRNA binding | 0.0036 | CRYZ, SRSF1, PCBP2, SNRNP70, G3BP2, PCBP1, HNRNPD, CNBP, HNRNBAP, RPS13 |
| GO:0008320 protein transmembrane transporter activity | 0.0103 | TOMM40L, TOMM70, PEX14 |


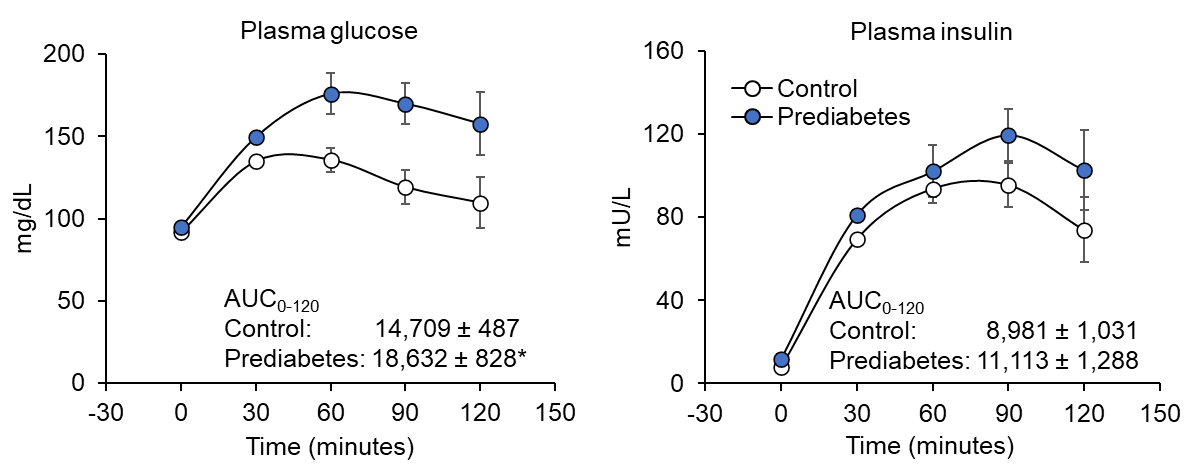


**Supplemental Figure 1 related to Table 1.** Plasma glucose (left) and insulin (right) concentrations during the oral glucose tolerance test in the prediabetic group (n=12) and the control group (n=18). Values are mean ± SEM. * Value significantly different from Control value, p <0.01.


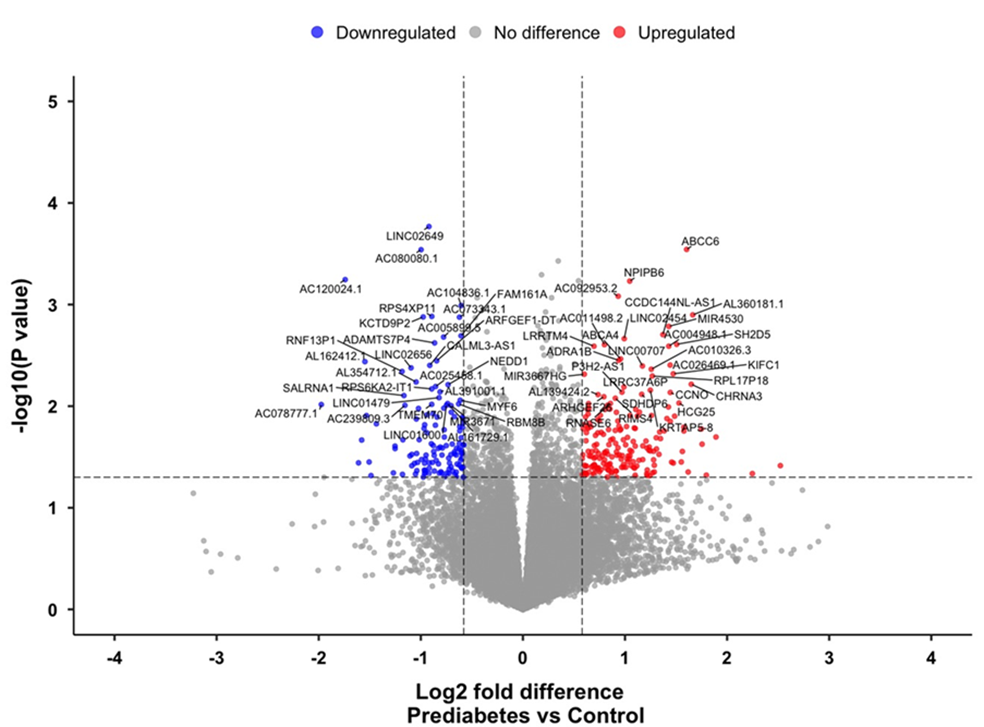


**Supplemental Figure 2 related to Figure 3.** Volcano plot of differences in muscle mRNA expression between the prediabetic group and the control group. The top 30 (among a total of 132) downregulated genes and the top 30 (among a total of 196) upregulated genes are shown with their abbreviated names. A total of 22,419 genes were monitored and differential expression was defined as a log2 fold-difference value ≥ 0.58 in conjunction with a -log10 p-value ≥1.3.


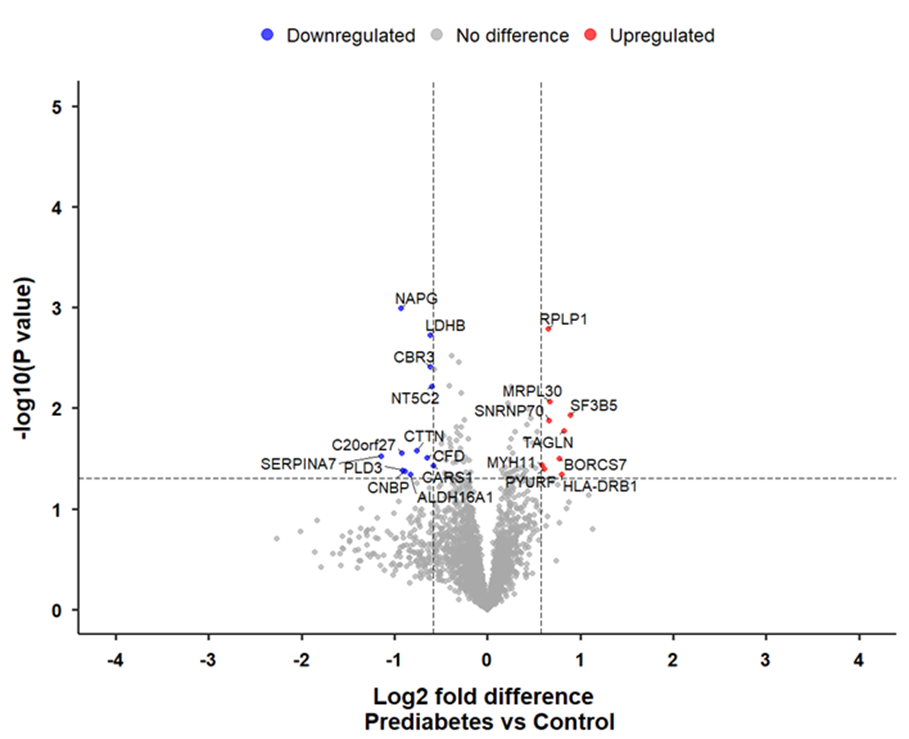


**Supplemental Figure 3 related to Figure 3.** Volcano plot of differences in muscle protein expression between the prediabetic group and the control group. A total of 2,900 proteins were monitored and the significantly down- and upregulated proteins are shown with their abbreviated names. Differential expression was defined as a log2 fold-difference value ≥ 0.58 in conjunction with a -log10 p-value ≥1.3.
